# The Diabetic Catalyst: A Rapid 12-Month Evolution of Hepatic Stiffness in a South Indian Cohort With Metabolic Dysfunction-Associated Steatotic Liver Disease

**DOI:** 10.7759/cureus.104756

**Published:** 2026-03-06

**Authors:** Roopa S Nibbaragandla, Pavan Sai Nelluri, SreeRam Thiriveedhi, Chaitanya L Narra, Anusha S Palagani, Ananya Ginjupalli, Chebrolu Harshitha Chowdary

**Affiliations:** 1 Medicine, Katuri Medical College and Hospital, Guntur, IND; 2 Community Medicine, Katuri Medical College and Hospital, Guntur, IND; 3 Surgery, Katuri Medical College and Hospital, Guntur, IND; 4 Internal Medicine, Katuri Medical College and Hospital, Guntur, IND; 5 Community Medicine, Psychiatry, General Medicine, Katuri Medical College and Hospital, Guntur, IND

**Keywords:** fibrosis‑4 (fib‑4), hepatic fibrosis, metabolic dysfunction, non-alcoholic fatty liver, types 2 diabetes

## Abstract

Background

Metabolic dysfunction-associated steatotic liver disease (MASLD) represents a significant clinical concern, particularly in populations where hepatic complications manifest at relatively low body mass index (BMI) thresholds. While type 2 diabetes mellitus (T2DM) is often associated with the progression of hepatic damage, there is a limited amount of prospective, longitudinal data comparing the specific rate of fibrosis development between diabetic and non-diabetic individuals in the South Indian clinical setting. This study aimed to monitor biochemical and radiological markers over a 12-month period to quantify the impact of T2DM on the temporal progression of hepatic fibrosis.

Methods

This prospective, longitudinal cohort study was conducted at a tertiary care academic hospital in South India from January to December 2024. A total of 126 participants with ultrasound-confirmed MASLD and a controlled attenuation parameter (CAP) >248 dB/m were initially enrolled. Following an attrition of 28 participants (22.2%) due to failure of follow-up, a final per-protocol analysis was conducted on 98 participants, divided into Group A (T2DM; n=49) and Group B (non-T2DM; n=49). Follow-up assessments were performed at baseline (M0), midpoint (M6), and endpoint (M12) using a double-look imaging strategy. Hepatic fat and stiffness were quantified using transient elastography (FibroScan®) to measure CAP and liver stiffness measurement (LSM), supplemented by the calculated Fibrosis-4 (FIB-4) index.

Results

At baseline, the diabetic cohort in Group A exhibited a significantly higher metabolic burden, including a higher mean BMI (27.2 ± 3.1 vs 25.1 ± 2.4 kg/m^2^; p=0.018) and higher hemoglobin A1C (HbA1c; 7.8 ± 1.2% vs 5.3 ± 0.3%; p<0.01) compared to Group B. Over the 12-month study period, Group A showed a linear and steady increase in mean LSM from 6.4 ± 1.6 kPa (7.1 ± 1.8 kPa at M6) to 7.8 ± 2.1 kPa (+1.4 kPa change; p<0.001), while Group B remained relatively stable (5.7 ± 1.3 to 5.9 ± 1.4 kPa; p=0.21). Additionally, 20.4% (n=10) of participants in Group A transitioned to a higher fibrosis stage, whereas only 4.1% (n=2) in Group B showed similar progression. Multivariable linear regression analysis confirmed that mean HbA1c remained a strong independent predictor of the increase in LSM (β = 0.45; p < 0.001) even after adjusting for baseline age and BMI. CAP scores indicating hepatic steatosis also worsened significantly in the diabetic cohort (292 ± 38 to 306 ± 42 dB/m; p=0.042).

Conclusion

The presence of T2DM accelerates the evolution of hepatic stiffness in South Indian MASLD patients, driving a substantial increase in fibrosis markers within a relatively short 12-month window. This progression underscores the unique metabolic vulnerability of the Indian population, where severe structural liver damage occurs at lower absolute body weights that already constitute clinical obesity (≥25 kg/m^2^) in this demographic. These findings emphasize the urgent need to integrate non-invasive screening tools such as FibroScan® and the FIB-4 index directly into primary diabetes care to facilitate early diagnosis and optimize the timing of specialized hepatology referrals.

## Introduction

The clinical landscape of chronic liver disease has undergone a paradigm shift over the last decade, with non-alcoholic fatty liver disease (NAFLD) emerging as the most prevalent cause of hepatic dysfunction globally. Recently renamed as metabolic dysfunction-associated steatotic liver disease (MASLD) to better reflect its metabolic roots, the condition encompasses a spectrum ranging from simple steatosis to non-alcoholic steatohepatitis (NASH), advanced fibrosis, and cirrhosis [1,2]. In India, the prevalence of NAFLD is alarmingly high, reflecting a unique Asian paradox where metabolic complications occur at lower body mass index (BMI) thresholds, often meeting regional criteria for clinical obesity (≥25 kg/m²) despite appearing non-obese by global standards. Current estimates suggest a national prevalence ranging from 9% to 32%, with urban clusters reporting figures as high as 55% [3,4]. As of 2026, India's status as a global epicentre for metabolic syndrome exacerbates this crisis, as nearly one-third of the adult population exhibits evidence of hepatic steatosis linked to rapid urbanization and sedentary lifestyles [5,6].

Pathophysiology: The multiple-hit hypothesis

The progression of NAFLD is driven by a complex, simultaneous series of metabolic insults known as the multiple-hit hypothesis. The primary driver is insulin resistance, which leads to an uncontrolled flux of free fatty acids (FFAs) from adipose tissue to the liver and increased de novo lipogenesis (DNL) [7]. This lipid overload results in lipotoxicity, where toxic lipid species induce endoplasmic reticulum stress and mitochondrial dysfunction, generating reactive oxygen species (ROS).

These processes trigger a pro-inflammatory response involving Kupffer cell activation and the release of cytokines such as tumor necrosis factor-alpha (TNF-α) and interleukin-6 (IL-6). Persistent inflammation eventually activates hepatic stellate cells (HSCs), which transition into myofibroblasts and deposit excessive extracellular matrix, leading to progressive hepatic fibrosis [8,9].

The interplay between T2DM and NAFLD

The relationship between T2DM and NAFLD is bidirectional and synergistic. T2DM acts as a potent catalyst, significantly accelerating the transition from simple steatosis to NASH and advanced fibrosis [10]. Clinical data from 2025 indicate that the prevalence of NAFLD among Indian patients with T2DM is strikingly high, often exceeding 75% [11]. Diabetes exacerbates hepatic fat accumulation, while the liver's insulin resistance, in turn, worsens glycaemic control, creating a vicious cycle of metabolic deterioration. Patients with both conditions face a drastically higher risk of liver-related mortality and cardiovascular events compared to those with either condition alone [12].

The knowledge gap

Despite the high prevalence of these co-morbidities, a significant gap exists in prospective, long-term data within the Indian population. Most existing literature is cross-sectional, providing only a snapshot of the disease state. There is a lack of head-to-head comparisons regarding the temporal rate of fibrosis progression between diabetic and non-diabetic cohorts. Furthermore, the clinical trajectory of MASLD at lower absolute body weights, a phenotype common in India where clinical obesity is defined at a much lower threshold (BMI ≥ 25 kg/m²), remains poorly understood in the context of T2DM [13]. Standardized screening protocols for liver fibrosis in primary diabetes care are also not yet universally implemented, leading to delayed diagnoses [14].

Rationale and objectives

Given the escalating dual burden of T2DM and NAFLD in India, it is critical to quantify how much faster the disease progresses in diabetic patients. This prospective cohort study aims to monitor biochemical and radiological markers over time to delineate the specific impact of T2DM on hepatic health. Understanding these differences is vital for developing targeted intervention strategies and optimizing the timing of specialized hepatology referrals.

## Materials and methods

Study setting and design

This prospective, longitudinal cohort study was conducted at a tertiary care academic hospital in South India. The study was designed to observe the naturalistic progression of NAFLD over a 12-month period, from January 2024 to December 2024.

Ethical considerations and consent

The study protocol received formal approval from the Institutional Ethics Committee (IEC), ensuring that all research procedures adhered to the ethical standards outlined in the 1964 Declaration of Helsinki and its subsequent amendments. In accordance with these standards, written informed consent was obtained from each participant prior to their enrollment in the study. Furthermore, to maintain participant privacy and data integrity, all sensitive information was anonymized using unique identification codes, thereby ensuring strict confidentiality throughout the entire study duration.

Selection criteria

The study population comprised individuals aged 18-65 years who were screened and subsequently assigned to one of two cohorts based on their glycemic status: the diabetic metabolic dysfunction-associated steatotic liver disease (MASLD) group (Group A) or the non-diabetic MASLD group (Group B). Participants were required to have a confirmed diagnosis of hepatic steatosis via transabdominal ultrasonography, characterized by increased echogenicity, which was further quantified using transient elastography (FibroScan®) [15]. A threshold for inclusion was set at a controlled attenuation parameter (CAP) score exceeding 248 dB/m [16]. Group A was defined by a documented history of T2DM according to the American Diabetes Association (ADA) guidelines, requiring a glycated hemoglobin (HbA1c) level ≥6.5% or the active use of glucose-lowering therapy [17]. In contrast, Group B participants were characterized by an HbA1c level < 5.7% and no prior history of dysglycemia.

To isolate the impact of metabolic dysfunction on hepatic health, several exclusion criteria were strictly applied. Individuals with significant alcohol consumption, defined as exceeding 20 g/day for females or 30 g/day for males, were excluded from the analysis [18]. Additionally, potential participants were screened for other causes of liver injury; those testing positive for viral hepatitis markers, such as hepatitis B surface antigen (HBsAg) or anti-hepatitis C virus (HCV) serology, or exhibiting evidence of autoimmune hepatitis, Wilson's disease, or alpha-1 antitrypsin deficiency, were omitted. Furthermore, the chronic use of steatogenic medications, including amiodarone, tamoxifen, methotrexate, or corticosteroids, precluded enrollment. Finally, individuals presenting with severe systemic illnesses, such as malignancy or end-stage renal disease, and those who were pregnant were excluded to ensure the safety and homogeneity of the study cohort.

Study population and follow-up

A total of 126 participants meeting the inclusion criteria were initially enrolled. During the 12-month follow-up period (M0-M12), 28 participants (22.2%) were lost to follow-up. Consequently, the final per-protocol analysis was conducted on 98 participants (Group A, n=49; Group B, n=49). Baseline characteristics of participants lost to follow-up were compared against those who completed the study using independent samples t-tests to evaluate for attrition bias. Based on an expected mean difference in LSM progression of 1.2 kPa, this sample size provided >80% power to detect significant differences at an alpha of 0.05.

Study protocol and assessment

The study followed a longitudinal design with participant assessments occurring at three distinct intervals: baseline (M0), a six-month mid-point (M6), and a 12-month endpoint (M12). During each clinical encounter, comprehensive anthropometric data were recorded, specifically focusing on BMI (calculated in kg/m^2^) and waist circumference. BMI classifications were strictly defined according to the consensus guidelines for Asian Indians: normal weight (18.0-22.9 kg/m²), overweight (23.0-24.9 kg/m²), and obese (≥ 25.0 kg/m²) [19]. These physical measurements were supplemented by a battery of biochemical analyses conducted through venous blood sampling. The laboratory parameters evaluated included liver function tests (LFTs), a full lipid profile, and HbA1c to monitor ongoing glycemic control.

To quantify the degree of hepatic fibrosis risk without the use of invasive biopsy procedures, the Fibrosis-4 (FIB-4) index was utilized as a non-invasive surrogate marker. This validated index integrates both clinical and biochemical data to provide a score that reflects the severity of liver architectural changes. The FIB-4 value for each participant at every follow-up interval was determined using the following formula in accordance with established clinical standards [15]:

$\text{FIB-4 Index} = \frac{\text{Age (years)} \times \text{AST (U/L)}}{\text{Platelet Count }(10^{9}/L) \times \sqrt{\text{ALT (U/L)}}}$

Imaging protocol

To ensure a rigorous longitudinal evaluation of hepatic structural changes, a dual-modality imaging protocol was implemented at baseline (M0), mid-point (M6), and 12-month endpoint (M12). The first component of this strategy involved transabdominal ultrasonography, which was conducted by a single, experienced radiologist using a 3.5 MHz curvilinear probe. This standardized approach allowed for the consistent grading of hepatic steatosis - ranging from Grade 0 to Grade III - while facilitating the monitoring of any emergent structural abnormalities throughout the study period.

The second component of the imaging protocol utilized transient elastography, specifically the FibroScan® 502 touch system (Echosens, Paris, France), to provide objective quantification of both hepatic fat and fibrosis. The CAP score was recorded to measure ultrasonic attenuation, providing a precise quantification of hepatic fat in units of dB/m [16]. Simultaneously, the liver stiffness measurement (LSM) was captured and recorded in kilopascals (kPa) to stage the severity of fibrosis. To maintain the diagnostic reliability and precision of these measurements, a strict acquisition protocol was followed; a minimum of 10 valid acquisitions was required for each participant, and a reading was only considered valid if the interquartile range to median ratio (IQR/median) remained below 30% [20].

The clinical indices and diagnostic tools utilized in this study, including the CAP, FIB-4 index, and the Asia-Pacific BMI classification, are established open-access clinical markers. Accordingly, no proprietary permissions or licensing agreements were required for their application in this research.

Statistical analysis plan

Data will be analysed using Statistical Product and Service Solutions (SPSS, version 27.0; IBM SPSS Statistics for Windows, Armonk, NY). Continuous variables will be compared using the unpaired t-test (between groups) and the paired t-test (within groups from M0 to M12). The difference in the proportion of participants experiencing a categorical shift to a higher disease stage between Group A and Group B was evaluated using the chi-square test. A p-value < 0.05 will be considered statistically significant. To account for baseline demographic disparities between the cohorts, multivariable linear regression analysis was performed. This model was utilized to identify independent predictors of hepatic stiffness progression while adjusting for potential confounders, specifically age and baseline BMI. Linear regression will be applied to assess if the mean HbA1c over 12 months independently predicts the change in LSM. To account for baseline demographic disparities between the cohorts, an analysis of covariance (ANCOVA) was conducted to compare the mean change in LSM (∆LSM) between Group A and Group B, utilizing baseline age and baseline BMI as covariates. Additionally, multivariable linear regression was applied to assess if mean HbA1c over 12 months independently predicts the change in LSM after adjusting for these same demographic confounders.

## Results

Participant flow and retention

A total of 126 participants were initially enrolled (Group A: n=63; Group B: n=63). Following the 12-month study period, 28 participants (22.2%) were lost to follow-up (Group A: n=14; Group B: n=14), resulting in a final analytical cohort of 98 participants (n=49 per group). The attrition was primarily due to failure of follow-up from the patients' end. A baseline comparison between the 28 participants lost to follow-up and the 98 retained participants showed no significant differences in age, BMI, mean HbA1c, or initial LSM (p>0.05), as analysed by independent samples t-tests. This suggests that data were missing at random, and the final cohort remained representative of the initial study population.

Baseline clinical and biochemical profiles

At the study's inception, the data revealed a distinct metabolic divergence between the two cohorts, which is characteristic of the metabolic trends observed in South Asian populations where complications manifest at relatively lower weight thresholds. As detailed in Table 1, the diabetic cohort (Group A) presented with a significantly higher mean BMI of 27.2 ± 3.1 kg/m^2^ (classified as clinically obese according to Asian Indian criteria: ≥ 25.0 kg/m²) compared to 25.1 ± 2.4 kg/m^2^ in the non-diabetic cohort (Group B), a difference that was statistically significant (t=3.78; p=0.018). This increased adiposity was further reflected in the mean waist circumference, which was notably higher in Group A at 98.2 ± 8.4 cm compared to 90.5 ± 7.6 cm in Group B (t=4.81; p<0.01). Consistent with their clinical classification, Group A demonstrated a mean glycated haemoglobin (HbA1c) of 7.8 ± 1.2%, indicating poor glycaemic control, whereas Group B maintained a mean level of 5.3 ± 0.3% (t=14.2; p<0.01).

**Table 1:** Baseline clinical and biochemical characteristics (M0) BMI: Body Mass Index (calculated as weight in kilograms divided by the square of height in meters, kg/m^2^); WC: Waist Circumference (measured in centimeters, cm); HbA1c: Glycated Hemoglobin (expressed as a percentage, %); ALT: Alanine Aminotransferase (measured in units per liter, U/L); AST: Aspartate Aminotransferase (measured in units per liter, U/L); FIB-4: Fibrosis-4 index (a non-invasive marker for liver fibrosis); CAP: Controlled Attenuation Parameter (measured in decibels per meter, dB/m); LSM: Liver Stiffness Measurement (measured in kilopascals, kPa)

Parameter	Group A (n=49)	Group B (n=49)	Test Statistic	p-value
Age (years)	53.4 ± 7.2	46.8 ± 8.1	t = 4.26	< 0.05
Gender (M:F)	28:21:00	27:22:00	chi-square = 0.04	0.84
BMI (kg/m^2^)	27.2 ± 3.1	25.1 ± 2.4	t = 3.78	0.018
Waist Circumference (cm)	98.2 ± 8.4	90.5 ± 7.6	t = 4.76	< 0.01
HbA1c (%)	7.8 ± 1.2	5.3 ± 0.3	t = 14.24	< 0.01
ALT (U/L)	48 ± 22	36 ± 15	t = 3.16	< 0.05
AST (U/L)	44 ± 18	32 ± 12	t = 3.89	< 0.05
FIB-4 Index	1.15 ± 0.38	0.88 ± 0.31	t = 3.85	< 0.05

Regarding liver-specific markers, the baseline levels of alanine aminotransferase (ALT) and aspartate aminotransferase (AST) were significantly elevated in the diabetic cohort relative to the non-diabetic cohort. Furthermore, the baseline risk for advanced liver disease was significantly higher in Group A, as evidenced by a mean FIB-4 index of 1.15 ± 0.38, whereas Group B presented with a lower mean index of 0.88 ± 0.31 (t=3.92; p<0.05). However, it must be acknowledged that this elevated baseline FIB-4 score in Group A is intrinsically driven in part by their significantly older mean age, as age serves as a direct numerator in the FIB-4 calculation algorithm. These biochemical findings suggest that the diabetic cohort possessed a higher metabolic burden and a more advanced stage of hepatic involvement at the time of enrolment.

Mid-point (six-month) trajectory analysis

In accordance with the study protocol, a mid-point assessment (M6) was performed to evaluate the temporal progression of hepatic markers. Analysis revealed that the increase in hepatic stiffness in the diabetic cohort (Group A) followed a steady, linear trajectory rather than an acute late-term spike. At the six-month interval, the mean LSM in Group A reached 7.1 ± 1.8 kPa, while the non-diabetic cohort (Group B) remained stable at 5.8 ± 1.3 kPa. Similarly, the mean CAP for Group A showed a mid-point increase to 298 ± 40 dB/m, compared to 260 ± 30 dB/m in Group B. These interim findings, summarized in Table 2, indicate a gradual evolution of hepatic involvement throughout the study duration.

**Table 2:** Mid-point (six-month) clinical and radiological findings LSM: Liver Stiffness Measurement (measured in kilopascals); CAP: Controlled Attenuation Parameter (measured in decibels per meter); T2DM: Type 2 Diabetes Mellitus

Parameter	Group A (n=49)	Group B (n=49)	Test Statistic	p-value
LSM (kPa)	7.1 ± 1.8	5.8 ± 1.3	t = 4.12	< 0.01
CAP (dB/m)	298 ± 40	260 ± 30	t = 5.34	< 0.01

End-of-study clinical and biochemical profiles (M12)

By the end of the 12-month period, the biochemical gap between the groups widened, particularly concerning markers of hepatic injury, as depicted in Table 3.

**Table 3:** Comparison of profiles at study endpoint (M12) LSM: Liver Stiffness Measurement (quantified in kilopascals, kPa); CAP: Controlled Attenuation Parameter (quantified in decibels per meter, dB/m); HbA1c: Glycated Hemoglobin (%); FIB-4: Fibrosis-4 Index; M0: Baseline Measurement; M12: 12-Month Endpoint Measurement

Parameter	Group A (n=49)	Group B (n=49)	Test Statistic	p-value
HbA1c (%)	8.1 ± 1.4	5.4 ± 0.4	t = 13.06	< 0.001
ALT (U/L)	54 ± 25	38 ± 16	t = 3.76	< 0.01
AST (U/L)	51 ± 20	34 ± 14	t = 4.87	< 0.01
FIB-4 Index	1.44 ± 0.52	0.92 ± 0.34	t = 5.86	< 0.001

Primary endpoints: Longitudinal hepatic progression

**Figure 1:**
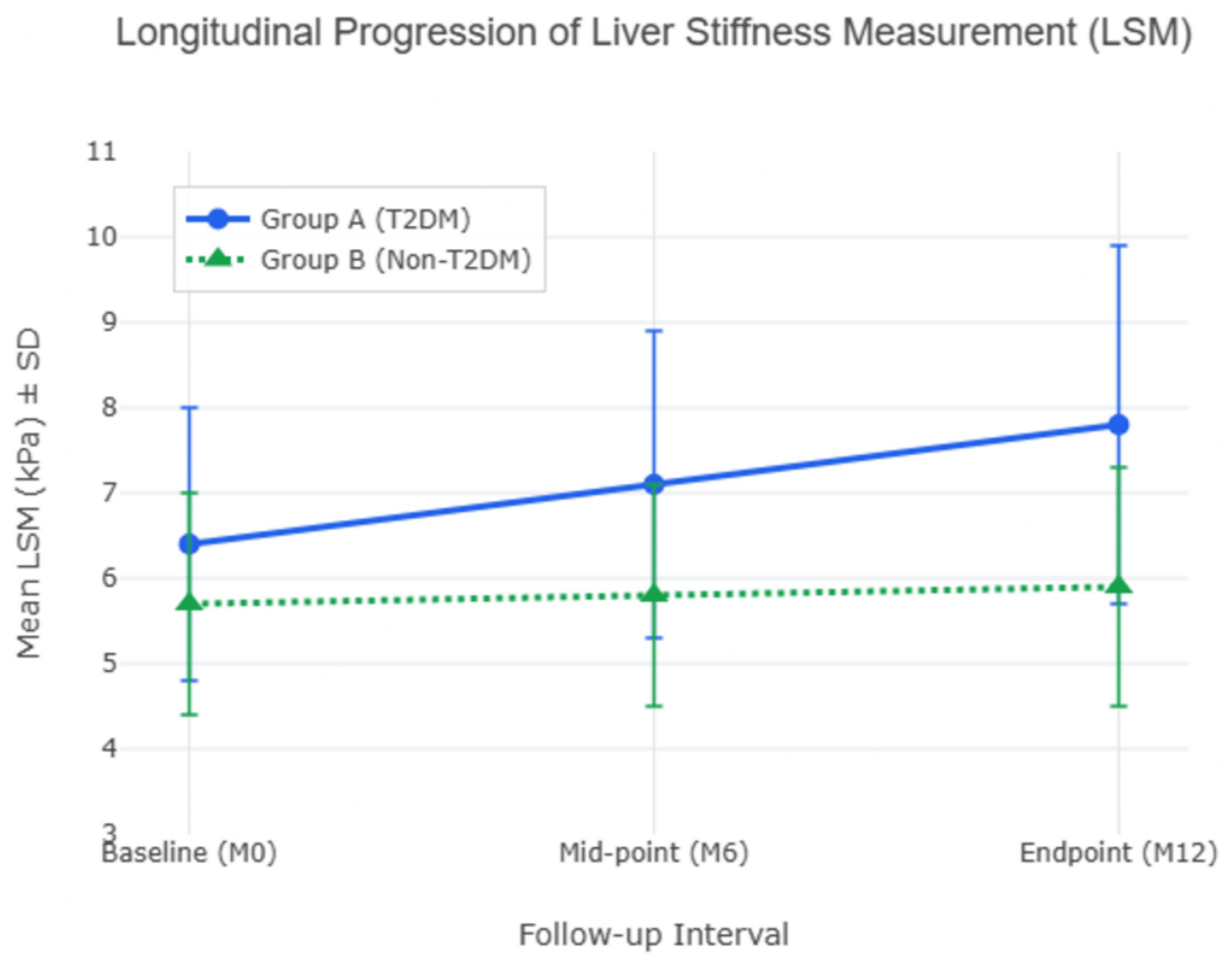
Twelve-month trajectory of hepatic stiffness progression Line plot detailing the temporal evolution of mean liver stiffness measurement (LSM, kPa) across three follow-up intervals: baseline (M0), mid-point (M6), and endpoint (M12). The diabetic cohort (Group A, blue circles) demonstrates a steady, linear progression of structural stiffness over the 12-month period (+1.4 kPa total change). In contrast, the non-diabetic cohort (Group B, green triangles) maintains a relatively stable trajectory (+0.2 kPa change). Error bars represent standard deviations (SD). Excel (Microsoft® Corp., Redmond, WA) was used in creating the figure for data visualization, and BioRender.com (BioRender, Ontario, Canada) was used for schematic illustrations.

The primary endpoint of the study focused on the 12-month longitudinal changes in hepatic health, quantified by LSM and CAP. This 12-month progression followed a steady, linear trajectory established at the six-month (M6) mid-point, as depicted in Table 2, which confirmed a gradual evolution of hepatic stiffness rather than an acute late-term spike. Transient elastography revealed that participants in the diabetic cohort (Group A) experienced a significant evolution of hepatic stiffness compared to their non-diabetic counterparts (Group B). Specifically, the mean LSM in Group A increased significantly from a baseline of 6.4 ± 1.6 kPa to 7.8 ± 2.1 kPa at the 12-month endpoint, representing a mean increase of +1.4 kPa (paired t=5.24; p<0.001). In contrast, the non-diabetic cohort demonstrated relative radiological stability, with the mean LSM moving from 5.7 ± 1.3 kPa to 5.9 ± 1.4 kPa, a marginal change of +0.2 kPa that failed to reach statistical significance (paired t=0.88; p=0.21). Crucially, an analysis of covariance (ANCOVA) confirmed that this accelerated LSM progression in the diabetic cohort remained highly statistically significant compared to the non-diabetic cohort even after adjusting for the confounding baseline effects of age and BMI (adjusted p<0.001). The LSM progression is visualized in Figure 1.

The clinical impact of this divergence was further evidenced by categorical stage shifts. Within the diabetic cohort, 20.4% (n=10) of participants transitioned to a more advanced fibrosis stage - such as progressing from F1 to F2 - whereas only 4.1% (n=2) of participants in the non-diabetic group exhibited a similar evolution of hepatic stiffness. This difference was statistically significant (chi-square=6.13; p=0.013), with an associated odds ratio (OR) of 5.9 (95% CI: 1.2-28.7), suggesting that the presence of T2DM significantly increases the risk of rapid fibrosis progression within a 12-month window.

Hepatic Steatosis Trends

Assessment of hepatic steatosis via CAP scores indicated a progressive accumulation of hepatic fat, primarily within the diabetic cohort. In Group A, the mean CAP score increased from 292 ± 38 dB/m at baseline to 306 ± 42 dB/m at the 12-month endpoint (paired t=2.06; p=0.042). Conversely, the non-diabetic cohort maintained a largely stable degree of steatosis, with CAP scores moving from 258 ± 28 dB/m to 262 ± 31 dB/m (paired t=0.56; p=0.58). These findings highlight that, while both groups began with confirmed steatosis, the diabetic environment served as a potent driver for both increasing fat volume and, more critically, the subsequent structural stiffening of the liver.

Correlation analysis

**Figure 2:**
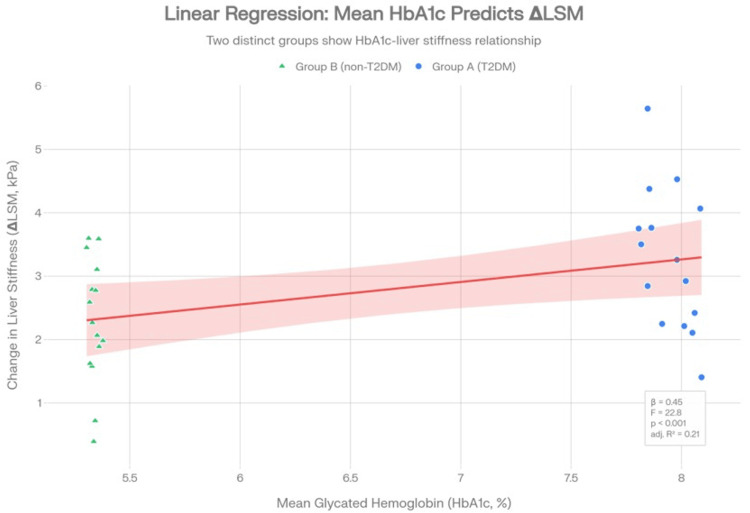
Relationship between glycemic control and hepatic stiffness progression Plot: Scatter plot of mean glycated hemoglobin (HbA1c %) versus 12-month change in liver stiffness measurement (∆ LSM, kPa). Plot: Scatter plot of the unadjusted correlation between mean HbA1c (%) and 12-month change in liver stiffness (ΔLSM, kPa). Cohorts: Group A (diabetic, blue circles); Group B (non-diabetic, green triangles). Fit: Regression line (red) with shaded 95% confidence interval. Adjustment: Multivariable model confirms HbA1c as an independent predictor even after adjusting for age and baseline BMI. Statistics: β=0.45, F=22.8, p<0.001, Adjusted R^2^=0.21 Excel (Microsoft® Corp., Redmond, WA) was used in creating the figure for data visualization, and BioRender.com (BioRender, Ontario, Canada) was used for schematic illustrations.

To address baseline differences in age and BMI between the cohorts, a multivariable linear regression model was employed. The adjusted model confirmed that mean HbA1c remained a strong independent predictor of the 12-month increase in LSM (β=0.45; F -statistic=22.8; p<0.001), indicating that glycemic control drives the evolution of hepatic stiffness independent of age or initial adiposity. These findings, detailing the impact of glycemic control on fibrosis progression, are visually represented in the scatter plot in Figure 2.

## Discussion

The clinical landscape of chronic liver disease has shifted significantly, with MASLD emerging as a global crisis. The results of this prospective 12-month cohort study reveal a critical and aggressive divergence in hepatic health between diabetic and non-diabetic individuals in South India. While the non-diabetic cohort (Group B) remained relatively stable, the diabetic group (Group A) experienced a mean increase in LSM of 1.4 kPa, rising from 6.4 ± 1.6 kPa to 7.8 ± 2.1 kPa. This evolution of hepatic stiffness challenges conventional timelines for fibrosis progression, suggesting that, in the presence of T2DM, hepatic health can decline significantly within a single year [21]. While a 1.4 kPa increase in LSM over 12 months may partially reflect active steatohepatitis or hepatocyte swelling rather than exclusive collagen deposition, the absence of significant ALT flares - with levels remaining below five times the upper limit of normal - suggests that this evolution of stiffness represents a clinically relevant progression of liver disease rather than a transient acute inflammatory event.

This trajectory is driven by the bidirectional and synergistic relationship between T2DM and MASLD, where chronic hyperglycaemia acts as a potent metabolic catalyst. According to the multiple-hit hypothesis, insulin resistance leads to an uncontrolled flux of free fatty acids to the liver and increased de novo lipogenesis [22]. This lipid overload induces lipotoxicity and oxidative stress, generating ROS that trigger pro-inflammatory responses and eventually activate HSCs into myofibroblasts. Our finding that mean HbA1c independently predicts the increase in LSM (β=0.45; p<0.001) reinforces that poor glycaemic control is not merely a comorbid state but a direct driver of fibrotic progression. Crucially, this association remained significant in our multivariable model even after adjusting for baseline age and BMI, confirming that the accelerated evolution of hepatic stiffness in the diabetic cohort was not merely a by-product of their older age or higher initial weight.

The observed progression in the diabetic cohort reinforces the Asian Paradox, where metabolic complications manifest at lower absolute BMI thresholds compared to Western populations. While the mean BMI of the diabetic cohort (27.2 kg/m^2^) might be classified merely as overweight by standard global WHO metrics, it falls firmly into the clinically obese category under Asian-Indian criteria (≥ 25.0 kg/m²). This highlights a critical reality of the South Indian context: severe metabolic complications and the rapid evolution of hepatic stiffness occur at much lower absolute body weights, emphasizing the danger of applying Western clinical thresholds to this demographic. Furthermore, the persistent elevation and increase in CAP scores in Group A indicate that diabetes exacerbates hepatic fat accumulation, creating a vicious cycle of metabolic disease progression.

When compared to existing literature, these results align with reports that the prevalence of NAFLD among Indian patients with T2DM often exceeds 75%. However, this study adds a vital temporal dimension often missing in cross-sectional research. The rapid 1.4 kPa increase in our cohort, compared to the stability in non-diabetics, highlights a critical knowledge gap regarding the speed of hepatic deterioration in this specific demographic. This rapid shift may also be influenced by the lack of universally implemented screening protocols in primary diabetes care, which often leads to delayed diagnoses of advanced fibrosis.

Several limitations must be acknowledged. The 22.2% attrition rate (28 lost out of 126) reflects the naturalistic challenges of conducting longitudinal research in a tertiary care setting. While sensitivity analysis showed no baseline bias, the per-protocol nature of the analysis may not capture the full diversity of the original population. Additionally, the study was conducted at a single academic hospital in South India, and the 12-month window is relatively short for assessing hard clinical outcomes such as cirrhosis.

Moving forward, these results emphasize the urgent need for a paradigm shift in diabetic management. Relying solely on liver enzymes is insufficient, as structural damage can progress even when biochemical markers are only mildly elevated. Integrating non-invasive screening tools such as the FIB-4 index or transient elastography directly into primary diabetes care could facilitate earlier interventions. Future multi-centric trials are warranted to delineate targeted intervention strategies and optimize the timing of specialized hepatology referrals to mitigate the escalating dual burden of T2DM and MASLD in India.

## Conclusions

This 12-month study confirms that T2DM significantly accelerates the progression of hepatic stiffness in South Indian MASLD patients, independent of baseline age and BMI. The diabetic cohort (Group A) exhibited a substantial mean LSM increase of 1.4 kPa (from 6.4 ± 1.6 to 7.8 ± 2.1 kPa), whereas the non-diabetic group remained relatively stable. Glycaemic control was identified as a potent, independent predictor of this rapid evolution of hepatic stiffness.

These findings underscore the Asian Paradox, where rapid disease progression occurs at lower absolute BMI thresholds, values that constitute clinical obesity according to Asian-Indian criteria. To improve clinical outcomes, non-invasive screening tools, such as transient elastography and the FIB-4 index, should be integrated into primary diabetes care to bridge the current diagnostic gap and facilitate timely intervention in this high-risk demographic.
